# Superior Foville syndrome due to pontine hemorrhage: a case report

**DOI:** 10.11604/pamj.2016.25.215.10648

**Published:** 2016-12-06

**Authors:** Daniel Gams Massi, Japhari Nyassinde, Mouhamadou Mansour Ndiaye

**Affiliations:** 1 Fann National Teaching Hospital-Cheikh Anta Diop University

**Keywords:** Superior Foville syndrome, hemorrhage, Sénégal

## Abstract

The Superior Foville Syndrome is a rare clinical feature of stroke or brain hemorrhage. Few cases have been reported worldwide particularly in Africa. We report the case of a 20 years old patient resident in Senegal with no known medical history. He was admitted on August 2015 in the Neurology Department of Fann National Teaching Hospital for an abrupt onset of left body side weakness, acute headaches and rotatory vertigo five days before admission. The physical examination found a superior Foville Syndrome. And the brain CT scan shown hemorrhage at the right inferior part of the pons compressing the fourth ventricle. No cause of this hemorrhage stroke has been found. Superior Foville syndrome is a rare clinical presentation of stroke and presented a rich semiological feature of the posterior cerebral fossa.

## Introduction

The Superior Foville Syndrome is a rare clinical feature of stroke or brain hemorrhage. It is characterized by ipsilateral sixth nerve palsy, facial palsy, facial hypoesthesia, peripheral deafness, Horner’s syndrome, contralateral hemiparesis, ataxia, pain, and thermal hypoesthesia, with lesions in the pontine tegmentum. Few cases have been reported worldwide particularly in Africa.

## Patient and observation

**Figure 1:**
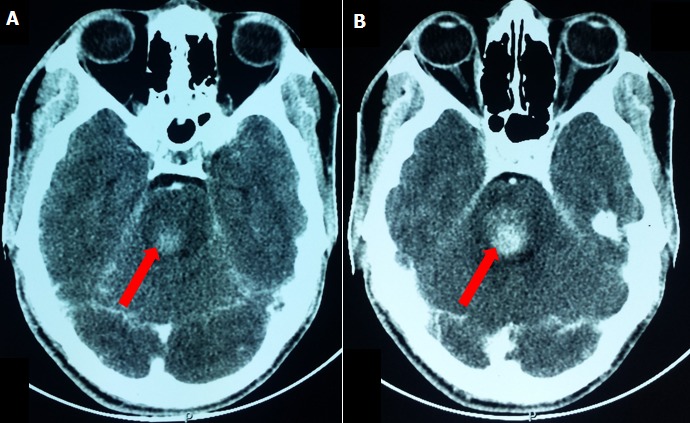
axial brain CT scan red arrow showing spontaneous hemorrhage located in the right pontine’s posterolateral part

We report the case of a 20 years old patient trader resident in Senegal with no known medical history. He was admitted on August 2015 in the Neurology Department of Fann National Teaching Hospital for an abrupt onset of left body side weakness five days before admission. That weakness was preceded by acute headaches and rotatory vertigo without nausea or vomiting. Physical examination found paralysis of right oculomotor movements, right facial palsy, and left hemiparesis sparing the face which constitute the superior pons type of Foville syndrome. The brain CT scan shown spontaneous hemorrhage in the right pontine’s posterolateral part compressing the fourth ventricle (Figure 1). Etiological investigations done to determine the cause of this hemorrhagic stroke remain non contributives. Patient received antalgic, anxiolytic, laxative and physiotherapy as treatment with clinical improvement. And he has been discharge 10 days later.

## Discussion

Foville’s syndrome was first described by the French anatomist and psychiatrist Achille-Louis François Foville in 1858 [1]. This syndrome suggests a lesion in lower pontine tegmentum [2]. Our patient presented hemorrhagic stroke of the right posterolateral part of the pons corresponding to the right lower tegmentum. It is characterized by ipsilateral sixth nerve palsy, facial palsy, facial hypoesthesia, peripheral deafness, Horner’s syndrome and contralateral hemiparesis, ataxia, pain, and thermal hypoesthesia [3]. The frequent causes found are infarction, hemorrhage, granuloma and tumor located in the pons [1–3].

## Conclusion

We report a case of superior Foville syndrome due to a pontine hemorrhage in a young patient. The clinical manifestations were well correlated with anatomical involvement. It is a rare clinical presentation of posterior cerebral fossa and represents a particular semiology feature of neurology. The treatment depends on the etiology found, so it is important to realize a complete work up.
